# The Interplay between Dietary Phosphorous, Protein Intake, and Mortality in a Prospective Hemodialysis Cohort

**DOI:** 10.3390/nu14153070

**Published:** 2022-07-26

**Authors:** Amanda R. Brown-Tortorici, Yoko Narasaki, Amy S. You, Keith C. Norris, Elani Streja, Rene Amel Peralta, Yalitzi Guerrero, Andrea Daza, Ria Arora, Robin Lo, Tracy Nakata, Danh V. Nguyen, Kamyar Kalantar-Zadeh, Connie M. Rhee

**Affiliations:** 1 Harold Simmons Center for Kidney Disease Research and Epidemiology, Division of Nephrology, Hypertension, and Kidney Transplantation, University of California Irvine, Orange, CA 92868, USA; amandab@uci.edu (A.R.B.-T.); ynarasak@hs.uci.edu (Y.N.); ssyou@hs.uci.edu (A.S.Y.); estreja@hs.uci.edu (E.S.); ramelper@hs.uci.edu (R.A.P.); yalitzig@hs.uci.edu (Y.G.); dazaa@hs.uci.edu (A.D.); 2riaarora@gmail.com (R.A.); robinhl@hs.uci.edu (R.L.); nakatat@hs.uci.edu (T.N.); danhvn1@hs.uci.edu (D.V.N.); kkz@uci.edu (K.K.-Z.); 2 Department of Medicine, David Geffen School of Medicine at UCLA, Los Angeles, CA 90095, USA; kcnorris@mednet.ucla.edu; 3 Tibor Rubin Veterans Affairs Medical Center, Long Beach, CA 90822, USA

**Keywords:** dietary phosphorus, dietary protein, end-stage renal disease, dialysis, mortality

## Abstract

(1) Background: Current dietary recommendations for dialysis patients suggest that high phosphorus diets may be associated with adverse outcomes such as hyperphosphatemia and death. However, there has been concern that excess dietary phosphorus restriction may occur at the expense of adequate dietary protein intake in this population. We hypothesized that higher dietary phosphorus intake is associated with higher mortality risk among a diverse cohort of hemodialysis patients. (2) Methods: Among 415 patients from the multi-center prospective Malnutrition, Diet, and Racial Disparities in Kidney Disease Study, we examined the associations of absolute dietary phosphorus intake (mg/day), ascertained by food frequency questionnaires, with all-cause mortality using multivariable Cox models. In the secondary analyses, we also examined the relationship between dietary phosphorus scaled to 1000 kcal of energy intake (mg/kcal) and dietary phosphorus-to-protein ratio (mg/g) with survival. (3) Results: In expanded case-mix + laboratory + nutrition adjusted analyses, the lowest tertile of dietary phosphorus intake was associated with higher mortality risk (ref: highest tertile): adjusted HR (aHR) (95% CI) 3.33 (1.75–6.33). In the analyses of dietary phosphorus scaled to 1000 kcal of energy intake, the lowest tertile of intake was associated with higher mortality risk compared to the highest tertile: aHR (95% CI) 1.74 (1.08, 2.80). Similarly, in analyses examining the association between dietary phosphorus-to-protein ratio, the lowest tertile of intake was associated with higher mortality risk compared to the highest tertile: aHR (95% CI) 1.67 (1.02–2.74). (4) Conclusions: A lower intake of dietary phosphorus was associated with higher mortality risk in a prospective hemodialysis cohort. Further studies are needed to clarify the relationship between specific sources of dietary phosphorus intake and mortality in this population.

## 1. Introduction

Hyperphosphatemia is a known predictor of mortality in hemodialysis patients [1,2,3]. Alterations in serum phosphorus levels have been related to subsequent disruptions of circulating parathyroid hormone levels and calcium homeostasis [4]. These derangements in mineral bone disease may subsequently lead to vascular calcification [5], left ventricular fibrosis and hypertrophy [6,7], and sudden cardiac death [8]. As hemodialysis does not sufficiently remove phosphorus towards recommended serum phosphorus target ranges [9,10], hyperphosphatemia is frequently observed among end-stage renal disease (ESRD) patients [2,11,12,13].

Current clinical practice guidelines advise ESRD patients treated with dialysis to consume a low phosphorus diet in order to mitigate hyperphosphatemia [14]. However, there is sparse evidence supporting these dietary recommendations. Among existing studies, there have been conflicting reports regarding the impact of dietary phosphorus restriction on outcomes of dialysis patients [15,16]. As lowering phosphorus intake has the potential to concomitantly reduce the consumption of heart-healthy macronutrients and micronutrients and heighten the risk of protein energy wasting (PEW) [17,18,19], there has been growing emphasis on dietary liberalization with concurrent use of phosphorus binder pharmacotherapies in the management of hyperphosphatemia in ESRD.

To address this gap in evidence, the overarching goal of this study was to evaluate the relationship between dietary phosphorus intake with survival in a well-characterized cohort of dialysis patients. In this study, we sought to examine the associations of specific dietary phosphorus indices, namely total absolute dietary phosphorus intake, dietary phosphorus scaled to 1000 kcal of energy intake, and phosphorus-to-protein ratio, with all-cause death risk in a multi-center prospective cohort of hemodialysis patients of diverse background. We hypothesized that a lower level of dietary phosphorus intake is associated with worse survival in this prospective hemodialysis cohort.

## 2. Materials and Methods

### 2.1. Study Population

The study population was comprised of a prospective cohort of hemodialysis patients from the *Malnutrition, Diet, and Racial Disparities in Chronic Kidney Disease* (MADRAD) cohort (ClinicalTrials.gov study: NCT01415570), an ongoing prospective study investigating the differential association between nutritional status and dietary factors across racial and ethnic subgroups. Among 1083 patients in the parent MADRAD cohort, patients in this MADRAD sub-study were recruited from 16 outpatient hemodialysis clinics across Southern California.

Patients were included provided that they were: (1) 18–85 years of age, (2) signed a consent form approved by the local institutional review board, and (3) received at least four consecutive weeks of in-center hemodialysis. Patients were excluded if they were: (1) unable to provide consent without a proxy, (2) had a life expectancy of less than six months, or (3) were actively receiving peritoneal dialysis treatment. Study approval was obtained from the Institutional Review Board of the University of California Irvine.

### 2.2. Exposure Ascertainment

The exposure of interest was dietary phosphorus consumption as measured by the Block food frequency questionnaire (FFQ). Patients were asked to fill out the Block FFQ at the time of study enrollment. The FFQ booklet contained 152 multiple-choice questions pertaining to 107 food items. The questionnaire asked how often each food/beverage had been consumed in the past several months, with responses ranging from “Never” to monthly frequencies, weekly frequencies, or daily. Then, based on the days they consume the food/beverage, patients were asked to fill in how much they consume using pictures of portion sizes. The United States Department of Agriculture Nutrient Database was used to analyze the nutrient content of foods/beverages in the FFQ.

In our primary analyses, we examined the association between daily absolute dietary phosphorus intake (mg/day), categorized as tertiles, and all-cause mortality risk. In the secondary analyses, we also examined the association between: (1) daily dietary phosphorus scaled to 1000 kcal of energy intake (mg/1000 kcal) and (2) daily dietary phosphorus-to-protein ratio (mg/g), categorized as tertiles with all-cause mortality risk. In the sensitivity analyses, we also examined the aforementioned dietary phosphorus indices categorized as quartiles. In order to model the association between dietary phosphorus ingestion as a continuous variable with all-cause mortality risk, we conducted restricted cubic spline analyses with knots corresponding to the 10th, 50th, and 90th percentiles of observed dietary phosphorus values.

### 2.3. Socio-Demographic, Comorbidity, and Dialysis Treatment Data

Baseline socio-demographic and comorbidity data were obtained at study entry. The definition of dialysis vintage was the period of time between the study entry date and the date of hemodialysis initiation. Standard dialysis laboratory measurements were conducted by outpatient dialysis clinics on a quarterly or monthly schedule using automated methods. Baseline values of routine laboratory tests, including serum phosphorus, serum albumin, normalized protein catabolic rate (nPCR), and serum creatinine, were examined in this study.

### 2.4. Body Anthropometry and Nutritional Measures

Body composition measurements were conducted while patients attended routine hemodialysis treatments. Body mass index (BMI) was calculated using the post-dialysis weight (kg) divided by height-squared (m^2^). Daily dietary intake of energy (kcal) and protein (g) were ascertained from the Block FFQ.

### 2.5. Outcome Ascertainment

The primary outcome of interest was all-cause mortality risk. At-risk time began the day after the baseline FFQ administration, and patients were censored for kidney transplantation, transfer to a non-affiliated outpatient dialysis unit or peritoneal dialysis, or at end of the study period (10 February 2018). At each semester, information regarding mortality, censoring events, and associated dates from the preceding six months was collected from event forms completed by the research coordinators and reviewed by the MADRAD Study nephrologists (K.K.-Z., C.M.R.).

### 2.6. Statistical Methods

We estimated the association between dietary phosphorus intake and all-cause mortality risk using Cox proportional hazard models with five incremental levels of covariate adjustment:

(1) *Unadjusted model*: No covariate adjustment;

(2) *Case-mix model*: Age, sex, race (Black vs. Non-Black), ethnicity (Hispanic vs. Non-Hispanic), and diabetes;

(3) *Expanded case-mix model*: Covariates in the case-mix model, as well as vintage, vascular access type, insurance, congestive heart failure, coronary artery disease, combined cardiovascular diseases, and BMI.

We a priori defined the expanded case-mix adjusted model as our primary model. To account for the possibility that various dietary and mineral bone disease covariates may be confounders vs. pathway intermediates of dietary phosphorus—mortality associations, we also conducted exploratory models that incrementally adjusted for laboratory, nutritional status, and mineral bone disorder markers using the following models:

(4) *Expanded case-mix + laboratory model*: Covariates in the expanded case-mix model, as well as dialysis adequacy (ascertained by spKt/V), serum albumin, nPCR, serum creatinine, and serum phosphorus;

(5) *Expanded case-mix + laboratory + nutrition*: Covariates in the expanded case-mix + laboratory model, as well as dietary energy intake, and dietary protein intake;

(6) *Expanded case-mix + laboratory + nutrition + mineral and bone disorder model*: Covariates in the expanded case-mix + laboratory + nutrition model, as well as serum calcium and parathyroid hormone levels.

While we had limited data on longitudinal phosphate binder use over time, we also conducted sensitivity analyses in which we incrementally adjusted for phosphate binder use within one-year of study entry: (7) *Expanded case-mix + laboratory + nutrition + mineral and bone disorder + medication model*: Covariates in the expanded case-mix + laboratory + nutrition + mineral and bone disorder model, as well as phosphate binder use vs. non-use.

We also conducted analyses examining the associations between the dietary phosphorus intake and all-cause death risk across clinically relevant subgroups according to socio-demographics, dialysis treatment characteristics, comorbidities, nutritional status, and mineral bone disorder status. There were no missing data for age, sex, race, ethnicity, vintage, dialysis access, insurance, diabetes, congestive heart failure, coronary artery disease, and combined cardiovascular disease; remaining covariates had <1% missing values except for BMI (3%), serum phosphorus (3%), serum albumin (3%), nPCR (3%), serum creatinine (5%), and spKt/V (6%). Analyses were conducted using STATA version 13.1 (Stata Corp., College Station, TX, USA) and SigmaPlot version 13 (Systat Software, SanJose, CA, USA).

## 3. Results

### 3.1. Cohort Description

Among 415 patients, the mean ± SD age of the cohort was 55 ± 15 years, among whom 45% were women, 36% were of Black race, 48% were of Hispanic ethnicity, and 55% had diabetes (Table 1). The mean and median (IQR) of daily dietary phosphorus intake in the cohort were 834 ± 678 and 695 (372, 1077) mg, respectively. The mean and median (IQR) of daily dietary protein intake in the cohort were 56 ± 47 and 45 (25, 73) mg, respectively. Compared to patients in the lowest absolute phosphorus consumption tertile, patients in the highest tertile were more likely to be male, non-Black, or Hispanic; had longer dialysis vintage; were less likely to have underlying diabetes; and were more likely to have combined cardiovascular diseases. Baseline characteristics stratified according to dietary phosphorus scaled to 1000 kcal of energy intake and dietary phosphorus-to-protein ratio are shown in Tables S1 and S2.

### 3.2. Absolute Dietary Phosphorus Intake and Mortality

**Figure 1:**
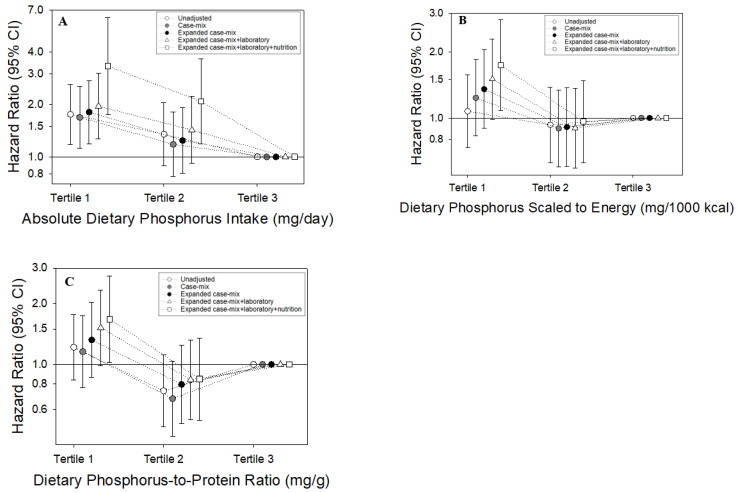
Association between daily absolute dietary phosphorus intake (**A**), phosphorus intake scaled to 1000 kcal of energy intake (**B**), and phosphorus-to-protein ratio (**C**) and all-cause mortality, respectively, among 415 MADRAD hemodialysis patients across tertiles.

Patients contributed a total of 1425 person-years of follow-up during which 151 death events were observed (Table S3). Median (IQR) at-risk time was 3.7 (1.9, 5.1) years. In the primary analyses of absolute dietary phosphorus intake categorized as tertiles, the lowest tertile of intake was associated with higher mortality risk (ref: highest tertile) in the expanded case-mix model: adjusted HR (aHR) (95% CI) 1.81 (1.19–2.75). Stronger magnitudes of risk were observed with incremental adjustment for laboratory, nutrition, and mineral bone disorder covariates: aHRs (95% CIs) 1.96 (1.27–3.02), 3.33 (1.75–6.33), and 3.35 (1.76–6.39) in the expanded case-mix + laboratory, expanded case-mix + laboratory + nutrition, and expanded case-mix + laboratory + nutrition + mineral bone disorder analyses, respectively (Figure 1A and Table S4). The findings were robust throughout the analyses, which were incrementally adjusted for baseline phosphate binder use: aHR (95% CI) in the expanded case-mix + laboratory + nutrition + mineral bone disorder + medication analyses (Table S5). Similar patterns were observed when analyzed by quartile (Figure S1A).

**Figure 2:**
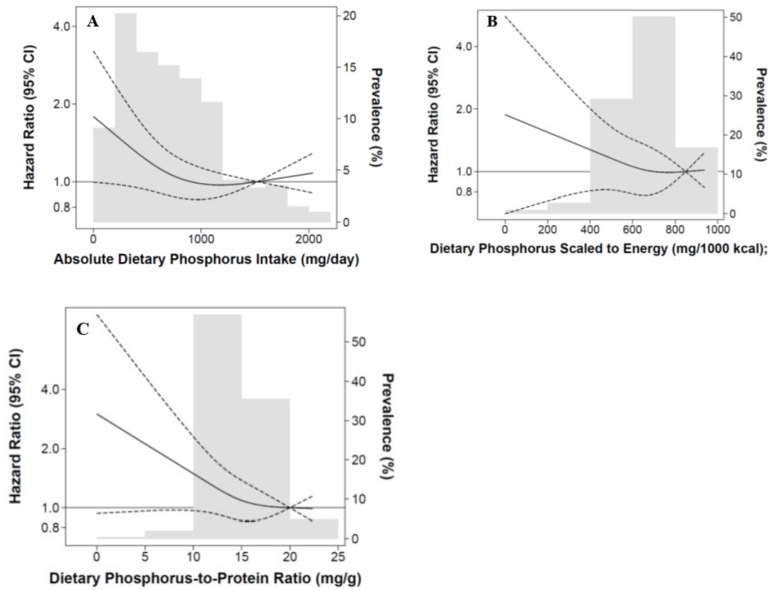
Association between baseline daily dietary phosphorus as a continuous variable and all-cause mortality among 415 MADRAD hemodialysis patients using restricted cubic spline analysis. Knots placed at the 10th, 50th, and 90th percentiles of observed values. (**A**) Absolute dietary phosphorus intake.(**B**) Dietary phosphorus scaled to energy. (**C**) Dietary phosphorus-to-protein ratio.

In restricted cubic spline analyses that examined the association between absolute phosphorus ingestion as a continuous variable and mortality risk with adjustment for expanded case-mix covariates, there was a monotonic increase in death risk with lower levels of intake (Figure 2A).

### 3.3. Dietary Phosphorus Intake Scaled to Energy Intake and Mortality

In the secondary analyses of dietary phosphorus scaled to 1000 kcal of energy intake (mg/1000 kcal) categorized as tertiles, the lowest tertile was associated with higher mortality risk in the expanded case-mix + laboratory + nutrition and expanded case-mix + laboratory + nutrition + mineral bone disorder models (ref: highest tertile): aHRs (95% CI) 1.74 (1.08–2.80) and 1.73 (1.07–2.80), respectively (Figure 1B and Table S4). A similar pattern was observed in analyses incrementally adjusted for baseline phosphate binder use (Table S5). Findings were also similar when analyzed by quartile (Figure S1B). In restricted cubic spline analyses that examined the association between dietary phosphorus scaled to 1000 kcal of energy intake as a continuous variable and mortality risk with adjustment for expanded case-mix covariates, there was a monotonic increase in death risk with lower levels of intake (Figure 2B).

### 3.4. Dietary Phosphorus-to-Protein Ratio and Mortality

In the secondary analyses of dietary phosphorus-to-protein ratio (mg/g) categorized as tertiles, the lowest tertile of intake was associated with worse survival in the expanded case-mix + laboratory + nutrition and expanded case-mix + laboratory + nutrition + mineral bone disorder models (ref: highest tertile): aHRs (95% CIs) 1.67 (1.02–2.74) and 1.65 (1.00–2.72), respectively (Figure 1C and Table S4). A similar pattern was observed in analyses incrementally adjusted for baseline phosphate binder use (Table S5). When analyzed by quartile, results trended towards higher mortality risk but did not achieve statistical significance (Figure S1C). In restricted cubic spline analyses that examined the association of dietary phosphorus-to-protein ratio as a continuous variable and mortality risk with adjustment for expanded case-mix covariates, there was a monotonic increase in death risk with lower levels of intake (Figure 2C).

### 3.5. Absolute Dietary Phosphorus Intake and Mortality across Clinically Relevant Subgroups

**Figure 3:**
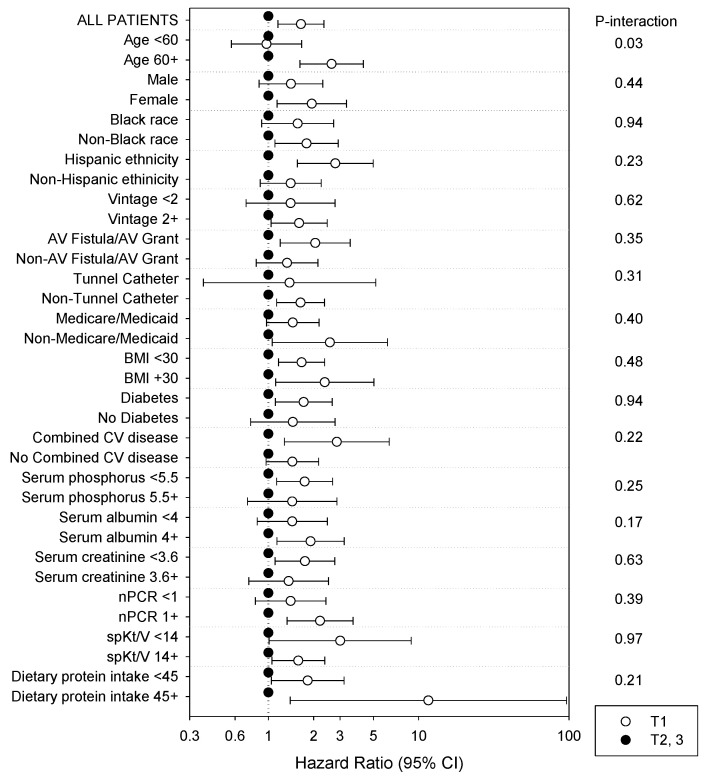
Association between lowest tertile of daily dietary phosphorus intake (ref: middle and highest tertiles) and all-cause mortality in 415 MADRAD hemodialysis patients across clinically relevant subgroups using expanded case-mix adjusted analyses.

We then examined associations of phosphorus consumption and survival across clinically relevant subgroups (mortality event number across subgroups). In the expanded case-mix analyses of absolute dietary phosphorus intake and death risk across clinically relevant subgroups, we detected effect modification on the basis of age (*p*-interaction = 0.03), such that the lowest tertile of intake was associated with higher mortality risk in those who were of older age (≥60 years old) but not in those of younger age (<60 years old): aHRs (95% CI) 2.63 (1.62, 4.29) and 0.97 (0.57, 1.66), respectively (Figure 3). We did not detect effect modification on the basis of sex, race, ethnicity, vintage, vascular access type, insurance status, BMI, diabetes status, cardiovascular disease status, serum phosphorus, serum albumin, serum creatinine, nPCR, spKt/V, of dietary protein intake levels. In all of the subgroups, the nominal HRs for mortality for the lowest tertile of absolute dietary phosphorus intake were >1, except among patients who were <60 years of age. Nominal associations were statistically significant among patients who were ≥60 years old, female, non-Black, or Hispanic; with vintage ≥ 2 years, with a AV fistula/graft, without tunneled catheter, or with a non-Medicare/Medicaid source as their primary insurance; with BMI < 30 or ≥ 30 kg/m^2^; with underlying diabetes or combined cardiovascular diseases; with serum phosphorus levels < 5.5 mg/dL, albumin levels ≥ 4 g/dL, creatinine < median of observed values, nPCR < 1 g/kg/day, or spKt/v < 1.4 or ≥ 1.4; or dietary protein intake < median or ≥ median levels of observed vales.

## 4. Discussion

In this multi-center prospective cohort of maintenance hemodialysis patients, we observed that those with lower dietary phosphorus intake had a higher mortality risk. These associations were robust across multiple secondary analyses examining various dietary phosphorus indices and sensitivity analyses examining potential confounders, including other nutritional factors, mineral bone disease indices, phosphate binder use, and clinically relevant subgroups.

Given the established association of high serum phosphorus levels with worse survival among hemodialysis patients, clinical practice recommendations often promote dietary phosphorus restriction as a means of mitigating hyperphosphatemia. However, according to Kidney Disease Improving Global Outcomes (KDIGO) guidelines, there is low-quality evidence in support of this practice therefore making it a weak recommendation [14]. By limiting phosphorus intake, patients may concomitantly reduce consumption of other critical nutrients such as protein, fat, complex carbohydrates, fiber, and various vitamins and minerals. Observational dietary studies of dialysis patients have suggested dietary restrictions are associated with decreased consumption of fruits and vegetables [18] and overall fiber [17]. Hence, the reduction in these heart-healthy nutrients may increase risk of cardiovascular morbidity and mortality [17].

Other observational studies have suggested that reduced dietary phosphorus intake may have adverse implications upon dialysis patient outcomes. In a secondary analysis of the HEMO study by Lynch and colleagues, prescribed low dietary phosphorus intake was not associated with a survival benefit among hemodialysis patients, suggesting that dietary phosphorus restriction may be potentially harmful [15]. In another study of over 30,000 hemodialysis patients by Shinaberger and colleagues, compared to patients with a six-month increase in serum phosphorus levels and dietary protein intake, those with increased serum phosphorus levels and decreased protein intake had 11% higher death risk, those with a concomitant decrease in both serum phosphorus and protein intake had 6% higher death risk, and those with decreased serum phosphorus but increased protein intake had 10% greater survival [19]. In contrast to these studies, a five-year prospective cohort of 224 hemodialysis patients by Noori et al. suggested that higher intake of dietary phosphorus and higher dietary phosphorus-to protein ratios were both associated with higher mortality risk [16].

In this contemporary, diverse cohort of hemodialysis patients who underwent protocolized FFQ’s, we also observed a significant relationship between lower phosphorus consumption and worse survival. Given the paramount importance of adequate nutritional status in hemodialysis patients, who are prone to hypercatabolism, protein and amino acid losses via dialysis, and protein-energy wasting, the risk of phosphorus restriction may outweigh its benefits, particularly if it leads to reduction in dietary protein intake and other heart-healthy nutrients [20]. In light of the mixed findings across various dialysis cohorts, further comparative effectiveness studies and clinical trials are needed to determine whether hyperphosphatemia management strategies that incorporate dietary phosphorus restriction vs. a more liberal diet with ample use of phosphorus binders with stronger binding capacities leads to improved nutritional, patient-centered, and hard outcomes in the ESRD population [3,21,22,23,24].

Another notable observation in our study was the differential association between dietary phosphorus intake and survival across hemodialysis patients of older vs. younger age. Whereas lower phosphorus intake was associated with a 2.6-fold higher death risk in patients ≥ 60 years of age, it was not associated with mortality in those < 60 years old. One potential explanation for these findings may be that inadequate nutritional status is more prevalent in elderly hemodialysis patients [25], who may be more vulnerable to protein-energy wasting. Another possible explanation may be related to the sources of phosphorus intake across age groups (i.e., organic vs. inorganic). Organic sources of phosphorus, such as plant-based and animal-based sources, may have absorption rates ranging from approximately <40% to 60%, whereas inorganic phosphorus, such as from additives, may have absorption rates ranging from 80–100% [26]. These inorganic phosphorus additives are commonly found in processed fast and convenience foods, and elderly CKD populations may consume higher amounts of convenience foods due to socio-economic and/or functional (i.e., activities of daily living) limitations [27]. Even with similar amounts of dietary phosphorus intake, the bioavailability of the phosphorus will dictate the effect on serum phosphorus and therefore mortality, and further study of the differential impact of dietary intake across age in ESRD are needed.

The strengths of our study include its availability of detailed patient-level data on sociodemographic, nutritional status, comorbidity, and laboratory data information from a contemporary, multi-center cohort of hemodialysis patients, as well as rigorous, protocolized collection of FFQ over time. However, there are several limitations that should be mentioned. First, responses to the FFQ are self-reported and may have been subject to under- or over-estimation of actual dietary intake. Second, the FFQ is unable to list every type of food typically eaten by hemodialysis patients, and therefore some phosphorus-containing foods may be under-reported in the questionnaire. Third, the FFQ analysis of nutrients does not differentiate between organic and inorganic phosphorus, which did not allow for discernment of type of phosphorus in our analyses. Fourth, due to data limitations we were not able to determine the relationships between dietary phosphorus intake with cause-specific mortality. Lastly, due to the observational nature of our study, we cannot confirm a causal association between dietary phosphorus intake and mortality.

## 5. Conclusions

In conclusion, in a prospective observational cohort of hemodialysis patients, we found that lower dietary phosphorus intake was associated with higher risk of all-cause mortality. However, further studies, including clinical trials, are needed to confirm findings, determine the impact of the differential sources of dietary phosphorus, and identify alternative strategies that can mitigate hyperphosphatemia without compromising nutritional status upon the health outcomes of the ESRD population.

## Figures and Tables

**Table 1:** Baseline characteristics of hemodialysis patients according to daily absolute dietary phosphorus intake categories.

	Overall	Dietary Phosphorus Intake (mg/Day)		
		Tertile 1	Tertile 2	Tertile 3
N (%)	415	138	137	140
Age (mean ± SD)	55 ± 15	57 ± 15	57 ± 13	53 ± 15
Male (%)	55	41	58	66
Black race (%)	36	35	34	40
Hispanic ethnicity (%)	48	46	47	52
Vintage (years, mean ± SD)	5 ± 4	4 ± 3	5 ± 4	5 ± 5
BMI (kg/m^2^, mean ± SD)	27.6 ± 6.6	27.4 ± 6.6	27.5 ± 6.0	27.9 ± 7.0
spKt/V	1.7 ± 0.3	1.7 ± 0.3	1.7 ± 0.3	1.7 ± 0.4
Dialysis access				
AV Fistula/Graft	47	39	50	53
Catheter	11	11	12	10
Unknown	41	50	37	37
Insurance				
Medicare/Medicaid	75	76	77	73
Private	11	13	12	9
Other	14	11	12	19
COMORBIDITIES				
Diabetes (%)	55	59	50	55
CHF (%)	8	9	7	9
CAD (%)	9	8	9	10
Combined CV disease (%)	17	16	15	19
LABORATORY RESULTS				
Serum phosphorus (mg/dL)	5.1 ± 1.5	5.1 ± 1.4	5.0 ± 1.3	5.1 ± 1.6
Serum albumin (g/dL)	4.0 ± 0.4	4.0 ± 0.3	4.0 ± 0.3	4.0 ± 0.4
nPCR (g/kg/day)	1.0 ± 0.3	1.0 ± 0.3	1.0 ± 0.3	1.0 ± 0.3
Serum creatinine (mg/dL)	9.7 ± 3.0	9.5 ± 3.1	9.6 ± 2.6	10.2 ± 3.1
DIETARY INTAKE				
Energy (kcal/day)	998 (566, 1527)	446 (302, 596)	1006 (842, 1252)	1790 (1398, 2373)
Protein (g/day)	45 (25, 73)	20 (14, 25)	45 (36, 55)	84 (69, 124)
MEDICATIONS				
Phosphate binder use (%)	93	92	94	93

## Data Availability

Data described in the manuscript, code book, and analytic code will be made available upon request pending application and approval by the corresponding author.

## Supplementary Materials

The following supporting information can be downloaded at: https://www.mdpi.com/article/10.3390/nu14153070/s1, Table S1: Baseline characteristics of hemodialysis patients according to daily dietary phosphorus intake scaled to 1000 kcal of energy intake; Table S2: Baseline characteristics of hemodialysis patients according to daily dietary phosphorus-to-protein intake (mg/g); Table S3: Association between dietary phosphorus intake and all-cause mortality in hemodialysis patients across tertiles (ref: highest tertile); Table S4: Association between dietary phosphorus intake and all-cause mortality in hemodialysis patients across quartiles (ref: highest quartile); Table S5: Association between lowest tertile of dietary phosphorus intake (ref: middle and highest tertiles) and all-cause mortality in hemodialysis patients across clinically relevant subgroups using expanded case-mix adjusted analyses; Figure S1: Association between daily absolute dietary phosphorus intake (Panel A), phosphorus intake scaled to 1000 kcal of energy intake (Panel B), and phosphorus-to-protein ratio (Panel C) and all-cause mortality, respectively, among 415 MADRAD hemodialysis patients across tertiles.

## References

[1] Saleh T., Sumida K., Molnar M.Z., Potukuchi P.K., Thomas F., Lu J.L., Gyamlani G.G., Streja E., Kalantar-Zadeh K., Kovesdy C.P. (2017). Effect of Age on the Association of Vascular Access Type with Mortality in a Cohort of Incident End-Stage Renal Disease Patients. Nephron.

[2] Tentori F., Blayney M.J., Albert J.M., Gillespie B.W., Kerr P.G., Bommer J., Young E.W., Akizawa T., Akiba T., Pisoni R.L. (2008). Mortality risk for dialysis patients with different levels of serum calcium, phosphorus, and PTH: The Dialysis Outcomes and Practice Patterns Study (DOPPS). Am. J. Kidney Dis..

[3] Young E.W., Albert J.M., Satayathum S., Goodkin D.A., Pisoni R.L., Akiba T., Akizawa T., Kurokawa K., Bommer J., Piera L. (2005). Predictors and consequences of altered mineral metabolism: The Dialysis Outcomes and Practice Patterns Study. Kidney Int..

[4] Silver J., Naveh-Many T. (2013). FGF-23 and secondary hyperparathyroidism in chronic kidney disease. Nat. Rev. Nephrol..

[5] Jono S., McKee M.D., Murry C.E., Shioi A., Nishizawa Y., Mori K., Morii H., Giachelii C.M. (2000). Phosphate regulation of vascular smooth muscle cell calcification. Circ Res..

[6] Faul C., Amaral A.P., Oskouei B., Hu M.C., Sloan A., Isakova T., Gutiérrez O.M., Aguillon-Prada R., Lincoln J., Hare J.M. (2011). FGF23 induces left ventricular hypertrophy. J. Clin. Investig..

[7] Amann K., Tornig J., Kugel B., Gross M.L., Tyralla K., El-Shakmak A., Szabo A., Ritz E. (2003). Hyperphosphatemia aggravates cardiac fibrosis and microvascular disease in experimental uremia. Kidney Int..

[8] Pun P.H., Horton J.R., Middleton J.P. (2013). Dialysate Calcium Concentration and the Risk of Sudden Cardiac Arrest in Hemodialysis Patients. Clin. J. Am. Soc. Nephrol..

[9] Lowrie E.G., Lew N.L. (1990). Death risk in hemodialysis patients: The predictive value of commonly measured variables and an evaluation of death rate differences between facilities. Am. J. Kidney Dis..

[10] Narasaki Y., Rhee C.M. (2020). Dietary Therapy for Managing Hyperphosphatemia. Clin. J. Am. Soc. Nephrol..

[11] Slinin Y., Foley R.N., Collins A.J. (2005). Calcium, phosphorus, parathyroid hormone, and cardiovascular disease in hemodialysis patients: The USRDS waves 1, 3, and 4 study. J. Am. Soc. Nephrol..

[12] Dwyer J.P., Kelepouris E. (2022). New Directions in Phosphorus Management in Dialysis. J. Renal Nutr..

[13] Fishbane S.N., Nigwekar S. (2021). Phosphate Absorption and Hyperphosphatemia Management in Kidney Disease: A Physiology-Based Review. Kidney Med..

[14] Kidney Disease: Improving Global Outcomes (KDIGO) CKD-MBD Work Group (2009). KDIGO clinical practice guideline for the diagnosis, evaluation, prevention, and treatment of Chronic Kidney Disease-Mineral and Bone Disorder (CKD-MBD). Kidney Int. Suppl..

[15] Lynch K.E., Lynch R., Curhan G.C., Brunelli S.M. (2011). Prescribed Dietary Phosphate Restriction and Survival among Hemodialysis Patients. Clin. J. Am. Soc. Nephrol..

[16] Noori N., Kalantar-Zadeh K., Kovesdy C.P., Bross R., Benner D., Kopple J.D. (2010). Association of Dietary Phosphorus to Protein Ratio with Mortality in Hemodialysis Patients. Clin. J. Am. Soc. Nephrol..

[17] Khoueiry G., Waked A., Goldman M., El-Charabaty E., Dunne E., Smith M., Kleiner M., Lafferty J., Kalantar-Zadeh K., El-Sayegh S. (2011). Dietary intake in hemodialysis patients does not reflect a heart healthy diet. J. Ren. Nutr..

[18] Kalantar-Zadeh K., Kopple J.D., Deepak S., Block D., Block G. (2002). Food intake characteristics of hemodialysis patients as obtained by food frequency questionnaire. J. Ren. Nutr..

[19] Shinaberger C.S., Greenland S., Kopple J.D., Van Wyck D., Mehrotra R., Kovesdy C.P., Kalantar-Zadeh K. (2008). Is controlling phosphorus by decreasing dietary protein intake beneficial or harmful in persons with chronic kidney disease?. Am. J. Clin. Nutr..

[20] Kim J.C., Kalantar-Zadeh K., Kopple J.D. (2013). Frailty and Protein-Energy Wasting in Elderly Patients with End Stage Kidney Disease. J. Am. Soc. Nephrol..

[21] Kalantar-Zadeh K., Tortorici A.R., Chen J.L., Kamgar M., Lau W.L., Moradi H., Rhee C.M., Streja E., Kovesdy C.P. (2015). Dietary restrictions in dialysis patients: Is there anything left to eat?. Semin. Dialysis..

[22] Gutekunst L. (2016). An Update on Phosphate Binders: A Dietitian’s Perspective. J. Ren. Nutr..

[23] Forfang D., Edwards D.P., Kalantar-Zadeh K. (2022). The Impact of Phosphorus Management Today on Quality of Life: Patient Perspectives. Kidney Med..

[24] You A.S., Kalantar S.S., Norris K.C., Peralta R.A., Narasaki Y., Fischman R., Fischman M., Semerjian A., Nakata T., Azadbadi Z. (2022). Dialysis symptom index burden and symptom clusters in a prospective cohort of dialysis patients. J. Nephrol..

[25] Qureshi A.R., Alvestrand A., Danielsson A., Divino J.C., Gutierrez A., Lindholm B., Bergström J. (1998). Factors predicting malnutrition in hemodialysis patients: A cross-sectional study. Kidney Int..

[26] Cupisti A., Kalantar-Zadeh K. (2013). Management of Natural and Added Dietary Phosphorus Burden in Kidney Disease. Semin Nephrol..

[27] Winger R.J., Uribarri J., Lloyd L. (2012). Phosphorus-containing food additives: An insidious danger for people with chronic kidney disease. Trends Food Sci. Technol..

