# Supplementary material for: The Interplay between Dietary Phosphorous, Protein Intake, and Mortality in a Prospective Hemodialysis Cohort
Source: Nutrients. 2022 Jul 26;14(15):3070. doi: 10.3390/nu14153070 (PMC9330827; doi:10.3390/nu14153070)
Supplement: Supplementary file 1 [file nutrients-14-03070-s001.zip › nutrients-1783133-supplementary.pdf]

SUPPREMENTARY MATERIALS

LEGEND

Table S1. Baseline characteristics of hemodialysis patients according to daily dietary phosphorus intake scaled to 1000 kcal of energy intake.

Table S2. Baseline characteristics of hemodialysis patients according to daily dietary phosphorus-to-protein intake (mg/g).

Table S3. Association between dietary phosphorus intake and all-cause mortality in hemodialysis patients across tertiles (ref: highest tertile).

Table S4. Association between dietary phosphorus intake and all-cause mortality in hemodialysis patients across quartiles (ref: highest quartile).

Table S5. Association between lowest tertile of dietary phosphorus intake (ref: middle and highest tertiles) and all-cause mortality in hemodialysis patients across clinically relevant subgroups using expanded case-mix adjusted analyses.

Figure S1. Association between daily absolute dietary phosphorus intake (Panel A), phosphorus intake scaled to 1000 kcal of energy intake (Panel B), and phosphorus-to-protein ratio (Panel C) and all-cause mortality, respectively, among 415 MADRAD hemodialysis patients across tertiles.

Table S1. Baseline characteristics of hemodialysis patients according to daily dietary phosphorus intake scaled to 1000 kcal of energy intake.

	Overall	Dietary Phosphorus Intake (mg/1000 kcal)		
		Tertile 1	Tertile 2	Tertile 3
**N (%)**	415	138	138	139
**Age (mean ± SD)**	55±15	55±14	55±15	56±15
**Male (%)**	55	55	52	59
**Black race (%)**	36	33	43	32
**Hispanic ethnicity (%)**	48	40	46	60
**Vintage (years, mean ± SD)**	5±4	5±4	5±4	4±4
**BMI (kg/m2, mean ± SD)**	27.6±6.6	27.5±7.2	28.5±6.7	26.9±5.6
**spKt/V**	1.7±0.3	1.7±0.4	1.7±0.3	1.7±0.3
**Dialysis access**				
AV Fistula/Graft	47	49	54	40
Catheter	11	12	8	14
Unknown	41	40	38	46
**Insurance**				
Medicare/Medicaid	75	80	73	73
Private	11	9	13	12
Other	14	12	14	16
**COMORBIDITIES**				
**Diabetes**	55	46	51	66
**CHF**	8	5	10	10
**CAD**	9	12	9	6
**Combined CV disease**	17	15	20	16
**LABORATORY RESULTS**				
**Serum phosphorus (mg/dL)**	5.1±1.5	5.1±1.5	5.1±1.5	5.0±1.4
**Serum albumin (g/dL)**	4.0±0.4	4.1±0.3	4.0±0.3	4.0±0.4

**nPCR (g/kg/day)**	1.0±0.3	1.0±0.3	1.0±0.3	1.1±0.3
**Serum creatinine (mg/dL)**	9.7±3.0	9.9±2.9	9.9±3.0	9.5±2.9
**DIETARY INTAKE**				
**Energy (kcal/day)**	998(566,1,527)	891(480,1,512)	1,108(608,1,596)	1,078(676,1,466)
**Protein (g/day)**	45(25,73)	32(19,60)	46(27,78)	53(33,80)

BMI, body mass index; AV, arteriovenous; CHF, congestive heart failure; CAD, coronary artery disease; CV, cardiovascular; nPCR, normalized protein catabolic rate.

Table S2. Baseline characteristics of hemodialysis patients according to daily dietary phosphorus-to-protein intake (mg/g).

	Overall	Dietary Phosphorus Intake (mg/g)		
		Tertile 1	Tertile 2	Tertile 3
**N (%)**	415	138	138	139
**Age (mean ± SD)**	55±15	54±15	56±15	56±14
**Male (%)**	55	53	62	52
**Black race (%)**	36	41	46	22
**Hispanic ethnicity (%)**	48	29	41	75
**Vintage (years, mean ± SD)**	5±4	4±4	5±4	5±4
**BMI (kg/m2, mean ± SD)**	27.6±6.6	27.2±6.7	28.6±7.1	27.0±5.7
**spKt/V**	1.7±0.3	1.7±0.3	1.7±0.3	1.8±0.4
**Dialysis access**				
AV Fistula/Graft	47	49	47	46
Catheter	11	12	9	12
Unknown	41	38	43	42
**Insurance**				
Medicare/Medicaid	75	75	73	78
Private	11	14	11	9
Other	14	12	16	14
COMORBIDITIES				
**Diabetes**	55	53	51	60
**CHF**	8	7	9	10
**CAD**	9	11	9	8
**Combined CV disease**	17	16	16	19
LABORATORY RESULTS				
**Serum phosphorus (mg/dL)**	5.1±1.5	5.3±1.4	4.8±1.4	5.1±1.6
**Serum albumin (g/dL)**	4.0±0.4	4.0±0.3	4.0±0.4	4.0±0.3

**nPCR (g/kg/day)**	1.0±0.3	1.1±0.3	1.0±0.3	1.0±0.3
**Serum creatinine (mg/dL)**	9.7±3.0	10.0±3.0	10.0±2.9	9.2±2.9
**DIETARY INTAKE**				
**Energy (kcal/day)**	998(566,1,527)	1,107(641,1,701)	938(547,1,424)	998(632,1,464)
**Protein (g/day)**	45(25,73)	60(31,93)	46(24,67)	39(22,58)

BMI, body mass index; AV, arteriovenous; CHF, congestive heart failure; CAD, coronary artery disease; CV, cardiovascular; nPCR, normalized protein catabolic rate.

Table S3. Association between dietary phosphorus intake and all-cause mortality in hemodialysis patients across tertiles (ref: highest tertile).

**Dietary Phosphorus Intake (mg/day)**						
	**Unadjusted**	**Case-mix adjusted**	**Expanded case-mix adjusted**	**Expanded case-mix+laboratory adjusted**	**Expanded case-mix+laboratory+ nutrition adjusted**	**Expanded case-mix+laboratory+ nutrition+MBD adjusted**
	**HR (95% CI)**	**HR (95% CI)**	**HR (95% CI)**	**HR (95% CI)**	**HR (95% CI)**	**HR (95% CI)**
**Tertile 1**	1.76(1.18,2.62)	1.69(1.12,2.54)	1.81(1.19,2.75)	1.96(1.27,3.02)	3.33(1.75,6.33)	3.35(1.76,6.39)
**Tertile 2**	1.35(0.89,2.06)	1.18(0.77,1.81)	1.24(0.81,1.91)	1.43(0.92,2.23)	2.09(1.19,3.67)	2.10(1.19,3.71)
**Tertile 3**	Reference	Reference	Reference	Reference	Reference	Reference
**P-trend**	0.005	0.011	0.005	0.002	<0.001	<0.001
**Dietary Phosphorus Intake Scaled to 1000 kcal (mg/1000 kcal)**						
	**Unadjusted**	**Case-mix adjusted**	**Expanded case-mix adjusted**	**Expanded case-mix+laboratory adjusted**	**Expanded case-mix+laboratory+ nutrition adjusted**	**Expanded case-mix+laboratory+ nutrition+MBD adjusted**
	**HR (95% CI)**	**HR (95% CI)**	**HR (95% CI)**	**HR (95% CI)**	**HR (95% CI)**	**HR (95% CI)**
**Tertile 1**	1.07(0.73,1.57)	1.24(0.83,1.84)	1.35(0.90,2.04)	1.50(0.99,2.29)	1.74(1.08,2.80)	1.73(1.07,2.80)
**Tertile 2**	0.93(0.63,1.38)	0.90(0.60,1.34)	0.91(0.60,1.37)	0.90(0.59,1.37)	0.96(0.63,1.48)	0.98(0.63,1.51)
**Tertile 3**	Reference	Reference	Reference	Reference	Reference	Reference
**P-trend**	0.733	0.335	0.185	0.078	0.033	0.034
**Dietary Phosphorus-to-Protein Ratio (mg/g)**						
	**Unadjusted**	**Case-mix adjusted**	**Expanded case-mix adjusted**	**Expanded case-mix+laboratory adjusted**	**Expanded case-mix+laboratory+ nutrition adjusted**	**Expanded case-mix+laboratory+ nutrition+MBD adjusted**
	**HR (95% CI)**	**HR (95% CI)**	**HR (95% CI)**	**HR (95% CI)**	**HR (95% CI)**	**HR (95% CI)**
**Tertile 1**	1.22(0.84,1.77)	1.16(0.77,1.74)	1.32(0.86,2.03)	1.52(0.99,2.34)	1.67(1.02,2.74)	1.65(1.00,2.72)

**Tertile 2**	0.74(0.49,1.12)	0.68(0.44,1.04)	0.80(0.51,1.24)	0.84(0.53,1.32)	0.85(0.53,1.35)	0.84(0.52,1.34)
**Tertile 3**	Reference	Reference	Reference	Reference	Reference	Reference
**P-trend**	0.308~~775~~	0.436~~787~~	0.166~~767~~	0.045~~328~~	0.037~~375~~	0.044~~376~~

MBD, Mineral and bone disorder.

* Tertiles for dietary phosphorus correspond to <460, 463-<933, and 943-4992 mg/day, respectively.

** Tertiles for dietary phosphorus scaled to 1000 kcal of energy intake (mg/1000 kcal) correspond to <606, 607-<736, and 737-1153 mg/1000 kcal, respectively.

*** Tertiles for dietary phosphorus-to-protein ratio correspond to intake of <13.4, 13.5-<15.93, and 15.95-57.5 mg/g, respectively.

Formatted: Highlight

Table S4. Association between dietary phosphorus intake and all-cause mortality in hemodialysis patients across quartiles (ref: highest quartile).

Dietary Phosphorus Intake (mg/day)						
	Unadjusted	Case-mix adjusted	Expanded case-mix adjusted	Expanded case-mix+laboratory adjusted	Expanded case-mix+laboratory+ nutrition adjusted	Expanded case-mix+laboratory+ nutrition+MBD adjusted
	HR (95% CI)	HR (95% CI)	HR (95% CI)	HR (95% CI)	HR (95% CI)	HR (95% CI)
Quartile 1	1.64(1.04,2.59)	1.49(0.94,2.38)	1.65(1.02,2.69)	1.93(1.16,3.20)	3.20(1.44,7.15)	3.32(1.48,7.42)
Quartile 2	1.24(0.76,2.03)	1.02(0.62,1.68)	1.11(0.67,1.84)	1.24(0.74,2.09)	1.89(0.91,3.93)	1.90(0.91,3.97)
Quartile 3	1.36(0.85,2.18)	1.14(0.70,1.84)	1.20(0.73,1.95)	1.49(0.89,2.51)	1.99(1.05,3.75)	2.05(1.08,3.90)
Quartile 4	Reference	Reference	Reference	Reference	Reference	Reference
P-trend	0.055	0.131	0.062	0.029	0.01	0.009
Dietary Phosphorus Intake Scaled to 1000 kcal (mg/1000 kcal)						
	Unadjusted	Case-mix adjusted	Expanded case-mix adjusted	Expanded case-mix+laboratory adjusted	Expanded case-mix+laboratory+ nutrition adjusted	Expanded case-mix+laboratory+ nutrition+MBD adjusted
	HR (95% CI)	HR (95% CI)	HR (95% CI)	HR (95% CI)	HR (95% CI)	HR (95% CI)
Quartile 1	1.03(0.66,1.61)	1.18(0.74,1.87)	1.30(0.81,2.08)	1.51(0.93,2.46)	1.80(1.05,3.09)	1.80(1.05,3.11)
Quartile 2	0.98(0.63,1.54)	1.08(0.68,1.70)	1.10(0.69,1.77)	1.10(0.68,1.77)	1.22(0.74,2.02)	1.22(0.73,2.02)
Quartile 3	0.92(0.59,1.43)	0.89(0.57,1.39)	0.95(0.60,1.50)	0.97(0.61,1.55)	1.03(0.65,1.65)	1.03(0.64,1.64)
Quartile 4	Reference	Reference	Reference	Reference	Reference	Reference
P-trend	0.837	0.38	0.246	0.101	0.039	0.04
Dietary Phosphorus-to-Protein Ratio (mg/g)						
	Unadjusted	Case-mix adjusted	Expanded case-mix adjusted	Expanded case-mix+laboratory adjusted	Expanded case-mix+laboratory+ nutrition adjusted	Expanded case-mix+laboratory+ nutrition+MBD adjusted

	HR (95% CI)	HR (95% CI)	HR (95% CI)	HR (95% CI)	HR (95% CI)	HR (95% CI)
**Quartile 1**	1.00(0.65,1.54)	0.85(0.53,1.37)	0.99(0.60,1.63)	1.11(0.67,1.85)	1.11(0.62,1.98)	1.14(0.63,2.05)
**Quartile 2**	0.82(0.52,1.28)	0.67(0.42,1.07)	0.76(0.46,1.24)	0.89(0.54,1.46)	0.89(0.53,1.50)	0.89(0.53,1.51)
**Quartile 3**	0.67(0.42,1.06)	0.55(0.34,0.88)	0.63(0.39,1.04)	0.59(0.36,0.97)	0.57(0.34,0.96)	0.59(0.35,0.99)
**Quartile 4**	Reference	Reference	Reference	Reference	Reference	Reference
**P-trend**	0.775	0.787	0.767	0.328	0.375	0.376

MBD, Mineral and bone disorder

* Dietary phosphorus intake quartiles 1, 2, 3 and 4 correspond to dietary phosphorus intake of <370, 372-<695, 695-<1064, and 1077-4992 mg/day, respectively.

** Dietary phosphorus/1000 kcal quartiles 1, 2, 3 and 4 correspond to intakes of <573, 574-<674.8, 674.9-<774, and 777-1153 mg/1000 kcal, respectively.

*** Dietary phosphorus-to-protein ratio quartiles 1, 2, 3 and 4 correspond to intakes of <12.9, 12.9-<14.6, 14.6-<17.03, and 17.03-57.5 mg/g, respectively.

Table S5. Association between lowest tertile of dietary phosphorus intake (ref: middle and highest tertiles) and all-cause mortality in hemodialysis patients across clinically relevant subgroups using expanded case-mix adjusted analyses.

	DIETARY PHOSPHOROUS INTAKE		
**SUBGROUPS**	**Tertile 1**	**Tertiles 2+3**	**p**
**Age**			
<60 years	0.97(0.57,1.66)	Reference	0.03
≥60 years	2.63(1.62,4.29)		
**Sex**			
Female	1.94(1.14,3.31)	Reference	0.44
Male	1.41(0.87,2.30)		
**Race**			
Non-Black	1.79(1.11,2.91)	Reference	0.94
Black	1.57(0.90,2.72)		
**Hispanic ethnicity**			
Yes	2.79(1.56,4.98)	Reference	0.23
No	1.41(0.88,2.25)		
**Vintage (years)**			
<2	1.41(0.71,2.78)	Reference	0.62
≥2	1.60(1.04,2.46)		
**AV Fistula/AV Grant**	2.05(1.20,3.50)	Reference	0.35
Yes	1.33(0.83,2.13)		
No			
**Tunnel Catheter**			
Yes	1.38(0.37,5.18)	Reference	0.31
No	1.64(1.14,2.36)		
**Medicare/Medicaid**			
Yes	1.45(0.97,2.17)	Reference	0.4
No	2.57(1.06,6.21)		
**BMI (kg/m^2)**			
<30	1.66(1.17,2.37)	Reference	0.48
≥30	2.37(1.12,5.03)		
**Diabetes**			
Yes	1.72(1.11,2.65)	Reference	0.94
No	1.45(0.76,2.77)		
**Combined CV Disease**	2.85(1.28,6.37)	Reference	0.22

Yes<br>No	1.44(0.96,2.16)		
**Serum phosphorus (mg/dL)**<br><5.5<br>≥5.5	1.74(1.13,2.67)<br>1.44(0.73,2.86)	Reference	0.25
**Serum albumin (g/dL)**<br><4<br>≥4	1.44(0.84,2.46)<br>1.91(1.14,3.20)	Reference	0.17
**Serum creatinine (mg/dL)**<br><9.6<br>≥9.6	1.75(1.11,2.77)<br>1.36(0.74,2.51)	Reference	0.63
**nPCR (g/kg/day)**<br><1<br>≥1	1.41(0.82,2.41)<br>2.21(1.33,3.66)	Reference	0.39
**spKt/V**<br><1.4<br>≥1.4	3.00(1.01,8.93)<br>1.58(1.05,2.37)	Reference	0.97
**Protein (g/day)**<br><45 (median)<br>≥45 (median)	1.83(1.05,3.19)<br>11.61(1.40,96.39)	Reference	0.21

BMI, body mass index; AV, arteriovenous; CV, cardiovascular; nPCR, normalized protein catabolic rate.

Figure S1.

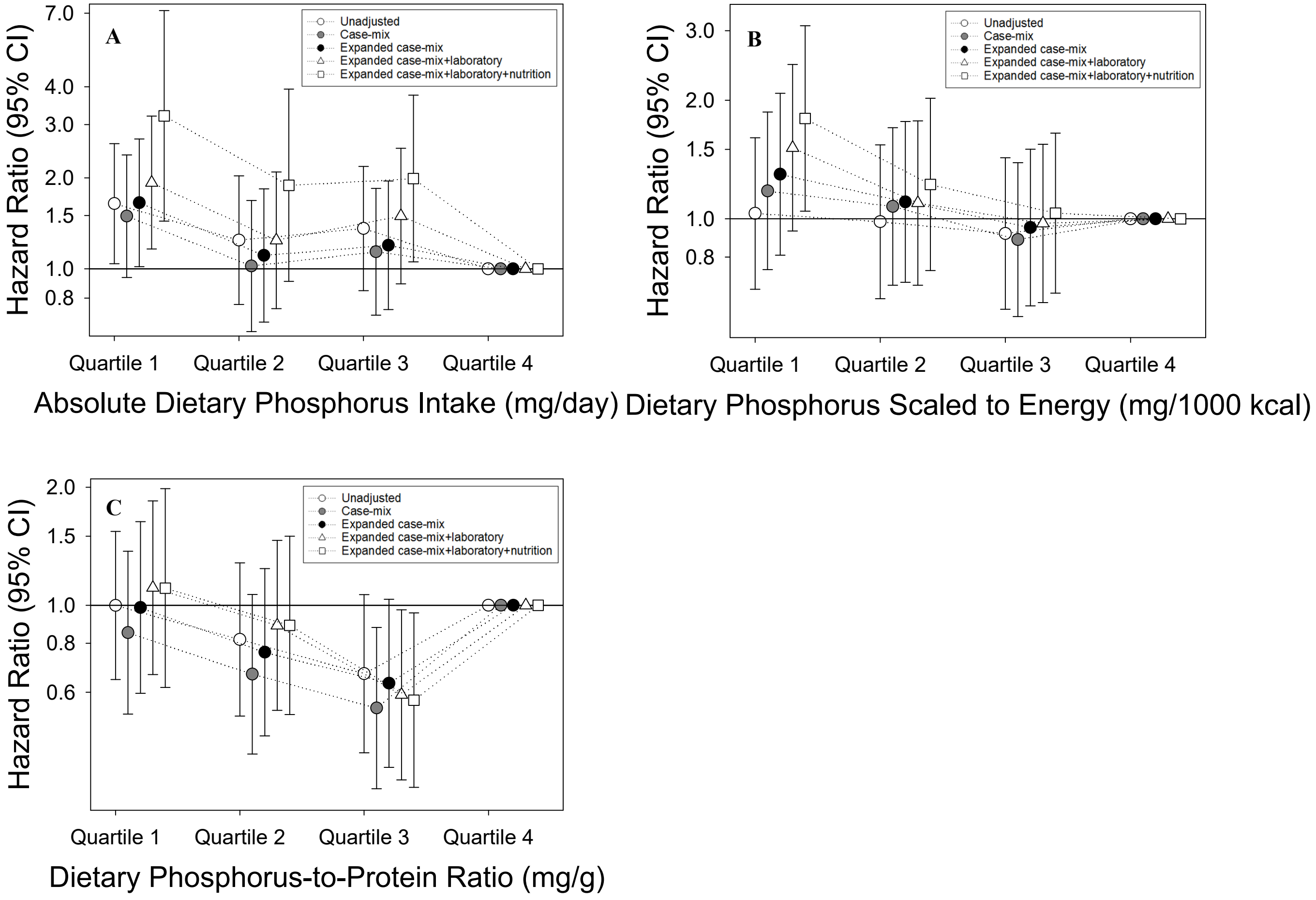
A
Unadjusted
Case-mix
Expanded case-mix
Expanded case-mix+laboratory
Expanded case-mix+laboratory+nutrition
Hazard Ratio (95% CI)
7.0
4.0
3.0
2.0
1.5
1.0
0.8
Quartile 1
Quartile 2
Quartile 3
Quartile 4
Absolute Dietary Phosphorus Intake (mg/day)
B
Unadjusted
Case-mix
Expanded case-mix
Expanded case-mix+laboratory
Expanded case-mix+laboratory+nutrition
Hazard Ratio (95% CI)
3.0
2.0
1.5
1.0
0.8
Quartile 1
Quartile 2
Quartile 3
Quartile 4
Dietary Phosphorus Scaled to Energy (mg/1000 kcal)
C
Unadjusted
Case-mix
Expanded case-mix
Expanded case-mix+laboratory
Expanded case-mix+laboratory+nutrition
Hazard Ratio (95% CI)
2.0
1.5
1.0
0.8
0.6
Quartile 1
Quartile 2
Quartile 3
Quartile 4
Dietary Phosphorus-to-Protein Ratio (mg/g)
